# Synergistic Effects of Plant Essential Oils and Extracts on Gut Microbiota in Rats

**DOI:** 10.3390/foods15020358

**Published:** 2026-01-19

**Authors:** Manasweeta Angane, Gunaranjan Paturi, Christine A. Butts, Siew Young Quek

**Affiliations:** 1 Food Science, School of Chemical Sciences, The University of Auckland, Auckland 1010, New Zealand; mang207@aucklanduni.ac.nz; 2 The New Zealand Institute for Bioeconomy Science Ltd., Auckland 1025, New Zealand; gunaranjan.paturi@plantandfood.co.nz; 3 The New Zealand Institute for Bioeconomy Science Ltd., Palmerston North 4472, New Zealand; chrissie.butts@plantandfood.co.nz; 4 New Zealand Centre of Research Excellence for Food Research, Riddet Institute, Palmerston North 4474, New Zealand

**Keywords:** essential oil, gut health, gut microbiome, natural food preservatives, plant extracts

## Abstract

The application of essential oils and plant extracts as natural food preservatives has gained increasing interest; however, their potential impacts on gut health and host physiology remain unknown. This study evaluated the effects of synergistic combinations of peppermint essential oil (EO) + thyme EO and peppermint EO + feijoa peel extract on gut microbiota composition and colonic morphology in a rat model. Sprague–Dawley rats were orally given the synergistic combinations daily for 28 days, and their effects were assessed using 16S rRNA gene sequencing of the caecum microbiota and histological analysis of proximal colon tissues. Alpha diversity metrics showed no significant differences (*p* > 0.05) between treatment and control groups, and beta diversity indicated no treatment-related shift in the bacterial communities. Taxonomic profiling at the phylum, family, and genus levels showed comparable relative abundances of dominant microbial taxa across all treatments, with no evidence of dysbiosis. Histological examination of proximal colon tissues revealed no significant changes in crypt depth between treated and control groups, confirming the absence of adverse morphological effects on the intestinal epithelium. The results of this study indicate that synergistic combinations of peppermint EO, thyme EO, and feijoa peel extract do not adversely affect the gut microbiota composition and colonic morphology in rats, thereby supporting their application as preservatives in foods.

## 1. Introduction

The dynamic host–gut microbiota association is highly susceptible to various environmental factors, particularly diet [1]. Biologically active substances, such as polyphenols, prebiotics, micronutrients, and vitamins, can target pathogenic bacteria and also modulate the immune system [2]. Many functional food ingredients, such as enzymes, probiotics [3,4], polyphenols [5], anthocyanins [6], flavonoids [7], prebiotics and dietary fibres [8,9], have been studied previously. Essential oils (EOs) and plant extracts are rich in aromatic compounds such as terpenes, terpenoids, polyphenols, flavonoids, and phenylpropenes [10,11]. They are extracted from plant parts such as the flowers, leaves, stems, roots, seeds, or fruits using methods such as steam distillation, extrusion, or solvent extraction. They have gained significant attention in cosmetics [12], medicine [13,14], and the food industry [15,16,17]. However, their effects may differ in living organisms due to various influencing factors, especially when combining multiple ingredients in a single formulation [1,18,19]. Several researchers have demonstrated the positive effects of EOs and plant extracts and their individual compounds in animal models, including anti-inflammatory properties, improvements in intestinal barrier function, and modulation of gut microbiota [20,21]. Other studies have explored their impact on blood metabolites, antioxidant, immunomodulatory properties and growth performance [2,22,23,24,25,26,27,28]. In animal science, EOs and extracts have emerged as potential alternatives to antibiotics due to their safety and minimal residual effects. Studies have demonstrated that EOs can enhance weight gain, improve resistance to infections in swine and poultry [29,30,31]. Moreover, EOs and their extracts possess antimicrobial and antiviral properties and have been investigated extensively for their ability to inhibit the growth of pathogenic viruses and bacteria [32,33].

To date, most research on EOs and extracts has focused on their antimicrobial activity in vitro [34,35]. However, there remains a lack of understanding regarding how they influence microbial communities in vivo. Our previous in vitro studies have assessed the antimicrobial activity of a range of EOs and plant extracts against common foodborne pathogens such as *Escherichia coli*, *Salmonella* Typhimurium, *Staphylococcus aureus*, *Bacillus cereus*, and *Listeria monocytogenes*. While individual EOs and extracts exhibited antimicrobial activity, their effectiveness varied across different bacterial species, with some EOs and extracts showing greater activity against Gram-positive bacteria and others being more effective against Gram-negative bacteria [36]. On this basis, the use of synergistic combinations has been proposed as a strategy to broaden the antimicrobial spectrum and enhance overall activity. The synergistic combinations of peppermint EO + thyme EO and peppermint EO + feijoa peel extract were selected based on our previous in vitro studies [36,37].

Previous studies have shown that the effects of EOs derived from culinary herbs and spices on the gut microbiota by increasing the beneficial bacteria such as *Lactobacillus* spp. and *Bifidobacterium* spp., and decreasing other bacterial populations belonging to phyla Firmicutes and Bacteroides [1,38]. In this study, we aim to assess the impact of EOs (peppermint and thyme) and a fruit peel extract (feijoa) on the gut microbiota composition and morphology in rats. The study specifically focuses on EOs and extracts sourced from New Zealand. Rats are used as an animal model to investigate the gut microbiota composition and establish correlations with human gut functions. To our knowledge, this is the first study to investigate the synergistic effects of peppermint EO, thyme EO, and feijoa peel extract in an animal model. The presence of phytochemicals and polyphenols in these oils and extracts has been suggested to modulate gut microbiota composition, including the promotion of beneficial bacteria and the suppression of pathogens. While we have not directly studied probiotic bacteria, this hypothesis is informed by previous studies [1,38].

## 2. Materials and Methods

### 2.1. Animals

The animal experimental procedures were approved by the AgResearch Grasslands Animal Ethics Committee (Ruakura) under the Animal Welfare Act 1999, New Zealand. Sprague-Dawley rats (6 weeks of age) weighing 200  ±  25 g were fed a commercial pelleted diet (LabDiet, Able Scientific, Auckland, New Zealand). The rats were housed in a controlled environment (22 ± 3 °C, 30–70% humidity) with a 12 h light/dark cycle. They were housed in same sex pairs and were provided with food and water ad libitum throughout the 4-week study period.

### 2.2. Administration of Essential Oils and Extracts

The EOs used in this study were peppermint and thyme purchased from Pure Ingredients, Glendene, Auckland, New Zealand, and feijoa peel extract prepared according to the method described earlier [36]. These EOs and extracts were selected based on their antimicrobial activity against the foodborne pathogens, *E. coli* and *L. monocytogenes* [37].

The rats were randomly assigned to three experimental treatments (control, peppermint EO + thyme EO and peppermint EO + feijoa peel extract) with 12 animals (6 males and 6 females) per treatment. The treatments were orally administered daily for 28 days. The control treatment was light olive oil (carrier oil). Treatment group 1 was peppermint EO + thyme EOs in the carrier oil. Treatment group 2 was peppermint EO + feijoa peel extract in the carrier oil. The specific doses of the EO and extract combinations used in the study for the peppermint EO + thyme EO and peppermint EO + feijoa peel extract combinations are described in Table 1.

The synergistic combination of EOs and extracts was prepared by mixing the specified volumes of individual components in a carrier oil (light olive oil) (Table 1). The control and EO treatments were administered daily to each rat via oral gavage, with the dose adjusted based on body weight (0.5 mL per 100 g body weight).

### 2.3. Body Weight Monitoring and Sample Collection

The rats were weighed and the body weights recorded twice weekly throughout the study period. At the end of the experiment (day 28), the rats were euthanised using CO_2_, and samples were collected for analysis. Caecum digesta was collected and stored at −80 °C for gut microbiota analysis. Proximal colon tissues were preserved in formalin for histological analysis.

### 2.4. Gut Microbiota Analysis

Bacterial genomic DNA from rat caecal contents was extracted using the QIAamp PowerFecal Pro DNA kit (Qiagen, Auckland, New Zealand). The quantity and quality of DNA were determined using the QIAxpert kit (Qiagen), and the DNA samples were sent to Massey Genome Service (Massey University, Palmerston North, New Zealand) for 16S rRNA gene sequencing of the V3–V4 hypervariable region. The polymerase chain reaction (PCR) barcoded fusion primers used were 16SF_V3 (5′-AATGATACGGCGA CCACCGAGATCTACAC-barcode-TATGGTAATTGGCCTACGGGAGGCAGCAG3′) and 16SR_V4 (5′-CAAGCAGAAGACGGCATACGAGAT-barcode-AGTCAGTCAGCCGGACTACHV GGGTWTCTAAT-3′) [39]. The amplicons were pooled in equal molarity and sequenced with a 2 × 250 base paired ends run on an Illumina MiSeq platform (Illumina Inc., San Diego, CA, USA) using version 3 chemistry. An Illumina PhiX Control v3 was included as the sequencing control.

### 2.5. Bioinformatics

The reads were processed using the standard Quantitative Insights into Microbial Ecology 2 (QIIME 2) v.2023.9 pipeline [40]. The reads were analysed by DADA2 methodology [41] in QIIME 2 to remove chimeric sequences and generate exact amplicon sequence variants. Taxonomic classifications were obtained using the embedded Naive Bayes fitted classifier trained on the GreenGenes2 full-length database. Alpha diversity (within a sample) was calculated using the Kruskal–Wallis test for diversity measures: Chao1, Pielou’s evenness, Faith’s phylogenetic diversity [PD], Observed operational taxonomic units (OTUs) and Shannon diversity. The beta diversity (between samples) was calculated using the Bray–Curtis dissimilarity, Jaccard index, and weighted and unweighted UniFrac distances. Permutational multivariate analysis of variance (PERMANOVA) was used to compare the differences between samples.

### 2.6. Histology

The colon tissues were excised from each rat and stored in 10% formalin until embedded in paraffin. The tissue processing was conducted using the Leica ASP300S Tissue Processor (Leica Biosystems, Nussloch, Germany). The tissues underwent sequential treatment in formalin, followed by ethanol solutions of 70% and 95%, and absolute ethanol, each for one hour. Subsequently, the tissues were treated with xylene for one hour. Following processing, the tissue samples were embedded in the Merck Histosec Pastilles^®^ (Darmstadt, Germany) Tissue Embedding Medium using the Leica Histo Embedder. Tissue blocks were sectioned into 5 μm-thick slices using the Leica RM2235 Manual Rotary Microtome. Three sections per slide were cut, with a 3 μm gap between each section. The tissue sections underwent staining using the Leica Autostainer XL Programme 4 and were stained with Alcian blue (pH 2.5) and haematoxylin and eosin (Sigma-Aldrich, Auckland, New Zealand) for microscopic examination [42]. Stained slides were dewaxed, and coverslips were placed on the slides using the Leica CV 5030 Robotic Coverslipper. Trajan 24 × 50 No. 1 coverslips were mounted onto the slides using Entellan^®^ Rapid Mounting Medium (Merck, Darmstadt, Germany) for Microscopy. An automated slide-scanner (Olympus SLIDEVIEW VS200, Tokyo, Japan) fitted with a CMOS colour camera (Olympus) was used to capture an image of the entire tissue section using a UPLXAPO 20×/0.8 NA objective lens. The colon tissue images were then used to measure the crypt depth using ImageJ (NIH, Bethesda, MD, USA) software version 1.50e (https://imagej.net/ij/, accessed on 18 April 2024) and to count the number of goblet cells per crypt [43].

### 2.7. Statistical Analysis

Rat body weight and colon morphology parameters were analysed using one-way analysis of variance followed by Tukey’s honestly significant difference (HSD at *p* = 0.05). The analyses were carried out using GenStat 22nd edition (VSN International, Hemel Hempstead, UK).

## 3. Results and Discussion

### 3.1. Effects of Essential Oil and Extract Combinations on Rat Body Weight

The average weekly weight gain for the rats ranged from 65 g to 80 g during week 1, 94 g to 113 g in week 2, 113 g to 136 g in week 3, and 130 g to 155 g in week 4. There were no significant differences (*p >* 0.05) in body weight between the control group and the treatment groups receiving the synergistic combinations of peppermint EO + thyme EO or peppermint EO + feijoa peel extract (Table 2). These findings show that the daily administration of the EOs and the extract combinations did not impact body weight in the rats and therefore did not affect the rats’ growth patterns over the 28-day study period.

### 3.2. Gut Microbiota

The gut microbiota analysis involved sequencing 16S rRNA amplicons from the samples and using QIIME2 for analysis. In total, approximately 11.8 million reads were generated across all samples, with individual samples ranging from 195,817 to 567,310 reads. The average number of reads per sample was 330,046, with a standard deviation of 71,548. This sequencing dataset provided a robust basis for subsequent microbiota analysis and diversity assessments. To standardise diversity analyses, all samples were rarefied to the same number of reads, using a threshold of 59,814 sequences (the number of sequences in the smallest sample).

**Figure 1:**
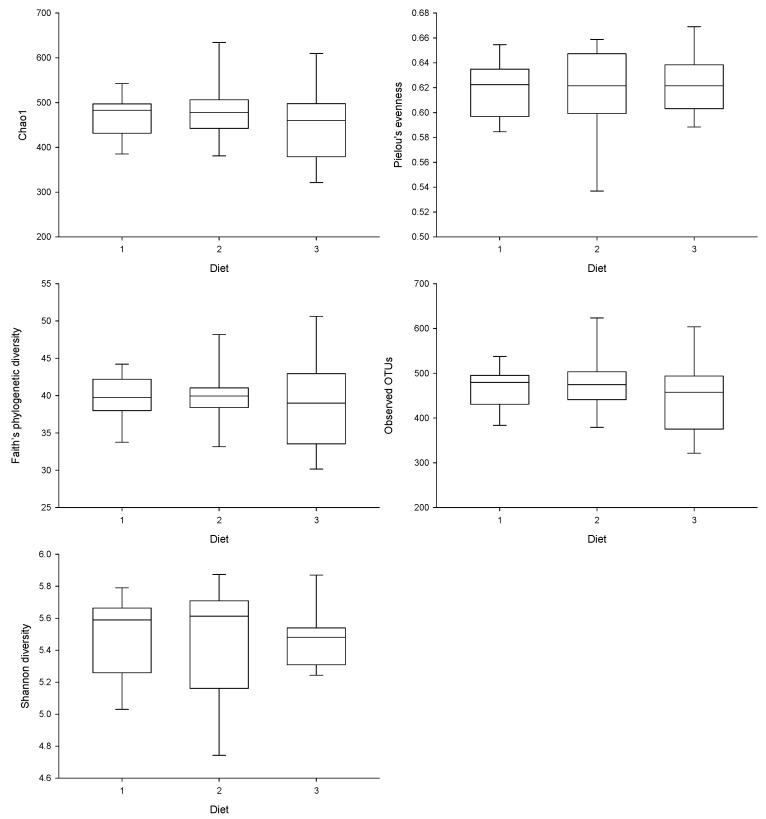
Alpha diversity analysis of rat gut microbiota following oral gavage with light olive oil (control) (1), peppermint + thyme (2) and peppermint + feijoa peel extract (3). Within-sample diversity was measured by Chao1, Pielou’s evenness, Faith’s phylogenetic diversity, Observed operational taxonomic units (OTUs) and Shannon diversity.

Based on the alpha diversity analysis, which assesses diversity within each sample based on diet categories, various metrics, including Chao1, Pielou’s evenness, Faith’s PD, Observed OTUs, and Shannon diversity, were employed. Each metric captures different aspects of sample complexity. The results indicate that there was no significant difference (*p* > 0.05) in alpha diversity, reflecting both richness and evenness, across the three treatments (Figure 1).

**Figure 2:**
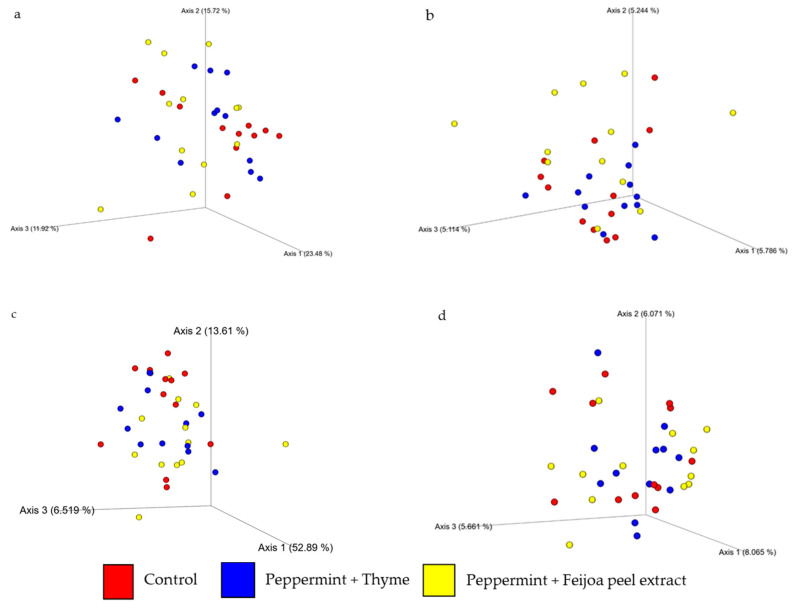
Principal Coordinates Analysis (PCoA) of beta diversity using Bray–Curtis dissimilarity (**a**), Jaccard index (**b**), weighted (**c**) and unweighted (**d**) UniFrac distances.

The beta diversity analysis assesses the diversity between samples using metrics such as the Bray–Curtis dissimilarity, Jaccard index, and weighted and unweighted UniFrac distances. These metrics determine whether the distances between samples within a diet group are more similar to each other than they are to samples from other diet groups. Based on the results (Figure 2), the rats fed the peppermint EO + feijoa peel extract exhibited higher beta diversity compared to the rats fed peppermint EO + thyme EO and the control across all metrics. The Jaccard index was statistically significant (PERMANOVA *p*-value = 0.014) between treatments. Pairwise comparisons with significant *q*-values showed that the rats fed the combination of peppermint EO + feijoa peel extract were distinct from the other two groups in terms of beta diversity (Figure 2). These differences in beta diversity suggest that specific EO and extract combinations had an impact on the gut microbiota composition. However, the extent to which these shifts affect overall gut health remains unknown and should be interpreted with caution, as no definitive patterns indicating either beneficial or adverse outcomes have been observed based solely on taxonomic composition. These results are consistent with previous studies. Li, Fu [44] found no significant difference in alpha diversity of the gut microbiota in response to treatment with pure EO compounds, and Qi, Mao [21] also reported no significant difference between control and cinnamaldehyde-treated groups in terms of alpha diversity, but observed alterations in beta diversity, indicating some changes in the gut microbial communities between the two groups.

**Figure 3:**
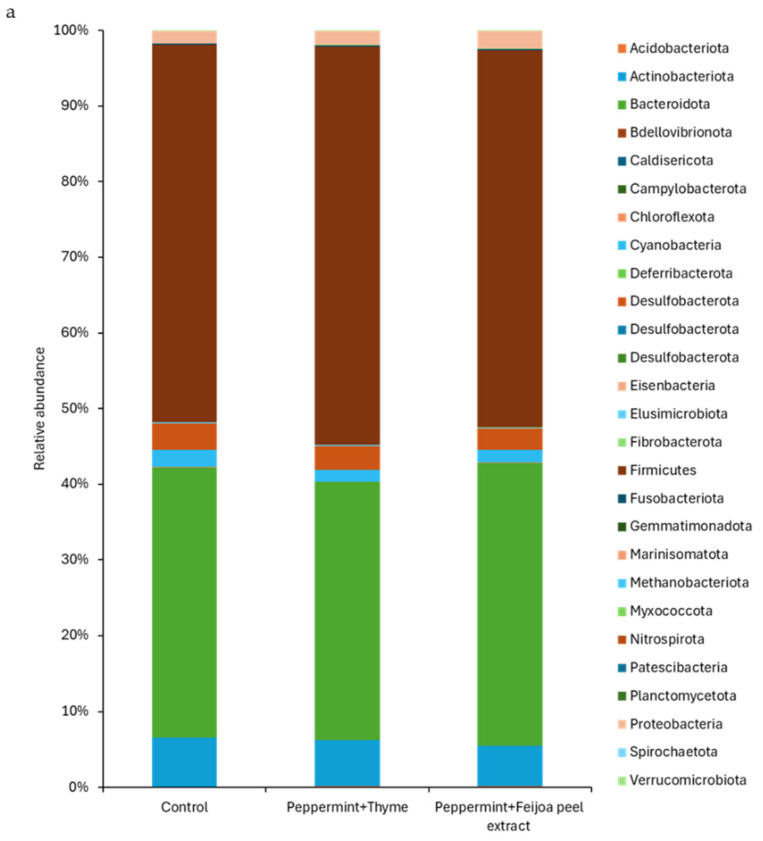

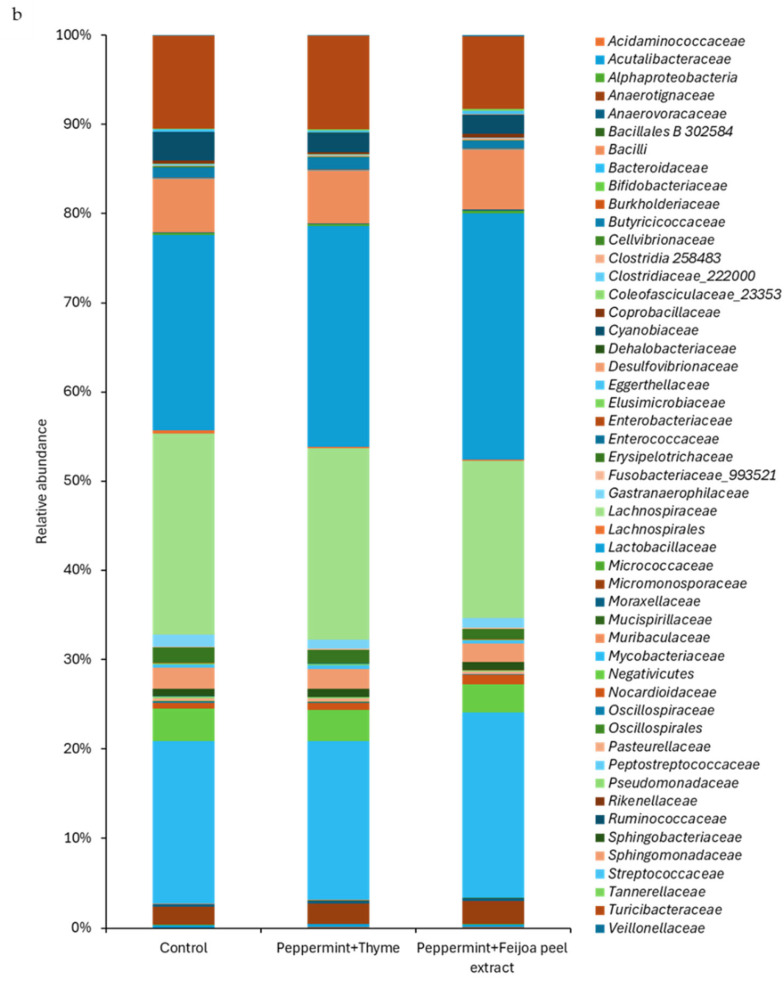

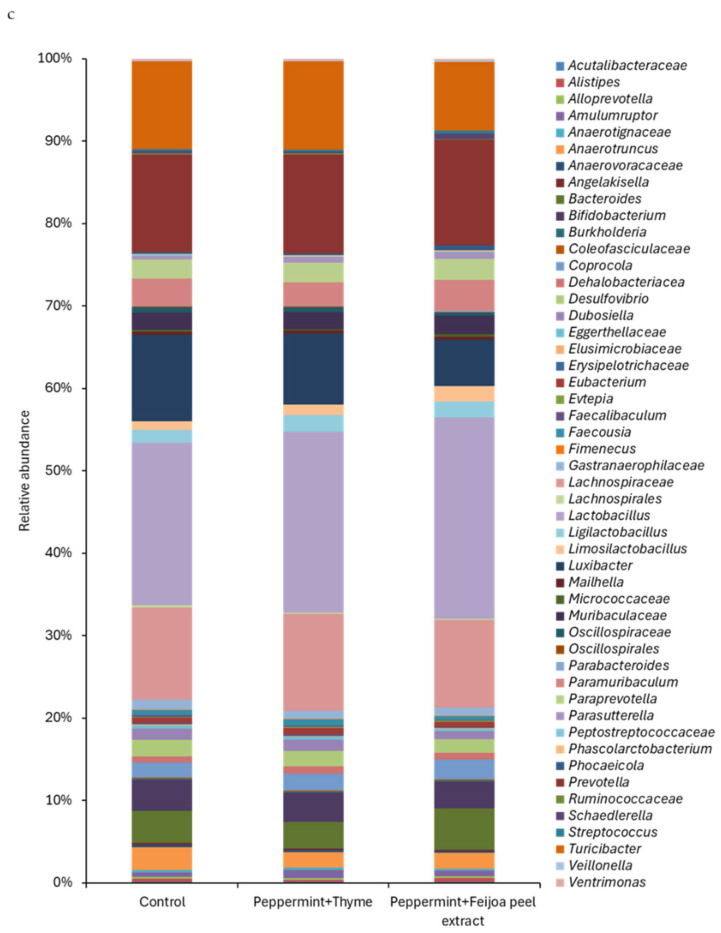
Relative abundance of bacteria in rat cecum at phylum (**a**), family (**b**), and genus (**c**) classification levels. At the phylum level, all the bacteria were presented. Whereas, for family and genus levels, the top 50 bacteria were presented.

The taxonomic composition of the gut microbiota at the phylum, family and genus levels was analysed across the three experimental groups (Figure 3). The analysis of the gut microbiota at the phylum level (Figure 3a) revealed that the predominant phyla across all treatment groups were Firmicutes and Bacteroidetes, followed by Actinobacteria. These results are consistent with previous studies [18,45] that have identified these phyla to be the dominant group in the gut microbiota of rats and piglets. The relative abundance of Firmicutes was highest in peppermint EO + thyme EO compared to the control group and the peppermint EO + feijoa peel extract. However, these differences were not statistically significant (*p* > 0.05). Similarly, the abundance of Bacteroidetes did not show significant differences among the diet groups, with values of 24,600 in the control group, 25,576 in the peppermint EO + thyme EO group, and 24,898 in the peppermint EO + feijoa peel extract group. The minor fluctuations observed in the relative abundances of Actinobacteria and Cyanobacteria were also not significant (*p* > 0.05).

The analysis of gut microbiota at the family level also showed that the administration of EOs and extracts did not result in significant changes in the composition of the gut microbiota in rats (Figure 3b). *Lactobacillaceae*, *Turicibacteraceae*, *Lachnospiraceae*, and *Bacteroidaceae* were the most abundant families across all groups. The most prominent trend was the increased abundance of *Lactobacillaceae* in the peppermint EO +thyme EO group and peppermint EO + feijoa peel extract group compared to the control group, but the difference was not significantly different (*p* > 0.05). This is consistent with the findings from other studies that reported an increase in *Lactobacillaceae* with the administration of specific EOs and extracts [1,25]. The other families, such as *Lachnospiraceae* and *Bacteroidaceae,* also showed no significant changes in their relative abundance across the different treatments (*p* > 0.05). The relative stability in the abundances of families such as *Bifidobacteriaceae*, *Muribaculaceae*, and *Gastranaerophilaceae* across all treatment groups suggests that these EOs and extracts did not disrupt the core microbial communities within the gut.

At the genus level (Figure 3c), the most prevalent genera were *Lactobacillus*, *Turicibacter*, *Luxibacter*, and *Prevotella*. *Lactobacillus* was more abundant in the peppermint EO + thyme EO group (22,743) and peppermint EO + feijoa peel extract group (21,118) compared to the control (19,270). *Turicibacter* exhibited the highest abundance in the peppermint EO + thyme EO group (11,160). *Luxibacter* was most abundant in the control group (10,241) and showed a marked decrease in the peppermint EO + feijoa peel extract group (4848). *Prevotella* levels were highest in the peppermint EO + thyme EO group (12,481). However, none of the differences were significantly different (*p* > 0.05).

The results of the taxonomic analysis indicate that the administration of peppermint EO + thyme EO and peppermint EO + feijoa peel extract did not cause significant changes in the gut microbial composition at the phylum, family, or genus levels. Therefore, these synergistic combinations did not notably impact the overall microbial diversity or the relative abundance of key microbial groups in the gut of Sprague-Dawley rats. In addition, the synergistic combinations of EOs and extracts did not cause major changes to the overall gut microbiota composition in rats. This is particularly important as it indicates that these EOs and extracts can be considered safe for use without causing dysbiosis, a disruption of the normal microbiota balance that could lead to adverse health effects.

In the present study, while trends such as increased relative abundance of *Lactobacillaceae* were noted in the treatment groups, these changes were not statistically significant (*p* > 0.05). This could be due to the specific EO-extract combinations and exposure duration. However, similar minor fluctuations were also observed in the control group, suggesting that the changes may reflect natural inter-individual or temporal microbiota dynamics rather than a direct effect of the treatments. Distinguishing treatment-driven effects from inherent microbial variability requires a longer study duration, greater replication, and possibly metagenomic confirmation to capture functional shifts.

Previous studies have shown that EOs and extracts can modulate gut microbiota. For instance, Ruzauskas, Bartkiene [38] reported an increase in probiotic bacteria in the caecum and colon of animals administered oregano extract, peppermint EO, and thyme EO in tablet form. Similarly, pomegranate peel extract was also reported to be selectively toxic against pathogenic bacteria [5,7]; and Li, Fu [44] found that carvacrol and thymol promoted the growth of *Bacillus*, *Lactobacillales*, *Streptococcaceae*, *Veillonellaceae*, and the genus *Megasphaera* in the colon. Our findings contrast with other studies [1,46] that reported significant changes in the gut microbiota composition due to the administration of EOs and extracts. However, the stability of the gut microbiota in our treatment groups, particularly the maintenance of Firmicutes and Bacteroidetes ratios, highlights that these EOs and extract combinations are safe.

**Figure 4:**
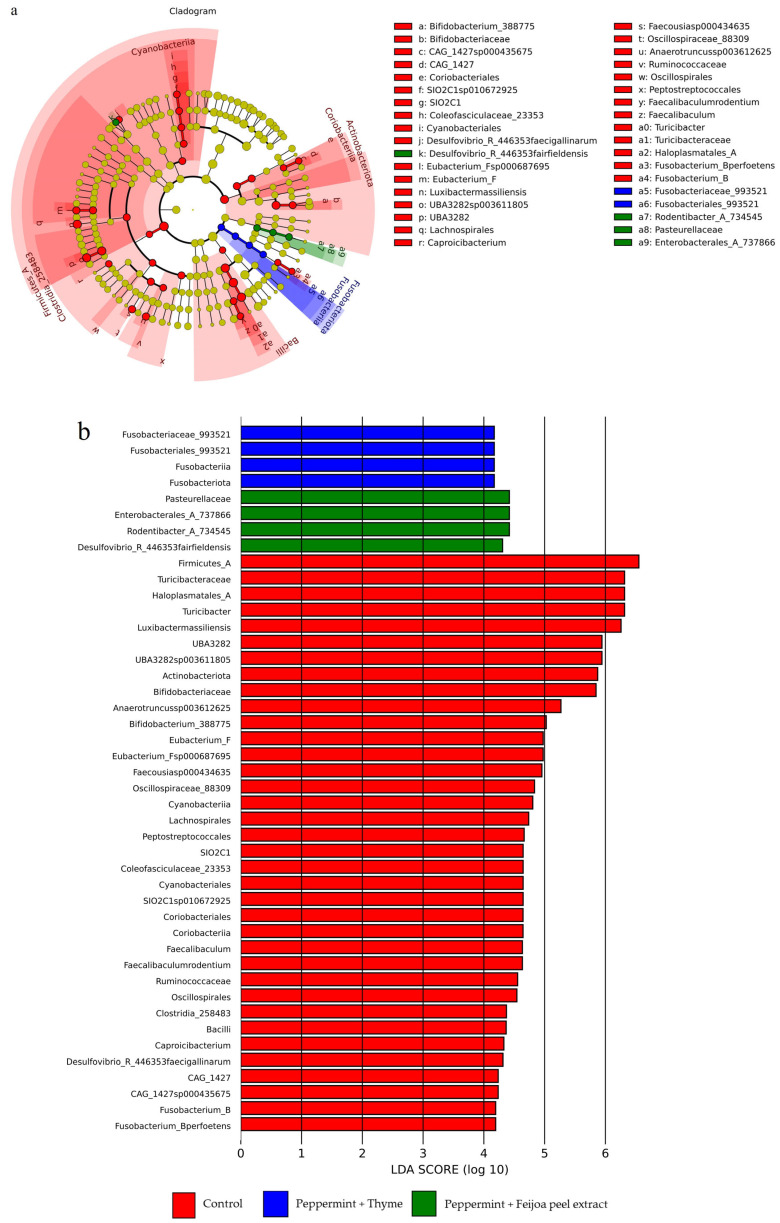
The cladogram (**a**) and linear discriminant analysis (LDA) score (**b**) obtained from LEfSe analysis of gut microbiota in rats across different experimental groups (control, peppermint + thyme and peppermint + feijoa peel extract).

The taxonomic analysis of gut microbiota was further examined using a cladogram and linear discriminant analysis (LDA) value distribution histogram to understand the differences in microbial composition among the treatments (control, peppermint EO + thyme EO, peppermint EO + feijoa peel extract). The cladogram illustrates the hierarchical structure of the microbial communities across the treatment groups (Figure 4a). The taxa that are significantly enriched in each group are highlighted by different colours: red for the control group, blue for the peppermint EO + thyme EO group, and green for the peppermint EO + feijoa peel extract group.

In the cladogram (Figure 4a), the control group shows a higher abundance of several taxa, including members of the families *Bifidobacteriaceae*, *Turicibacteraceae*, *Lachnospiraceae*, and the genus *Turicibacter*. These taxa are primarily associated with the phyla Firmicutes and Actinobacteriota. The peppermint EO + thyme EO group had a higher abundance of *Fusobacteriaceae* and *Fusobacteriales*, indicating a distinct microbial profile influenced by this treatment. Similarly, the peppermint EO + feijoa peel extract group showed a unique set of enriched taxa, particularly in the genus *Rodentibacter*, *Enterobacterales*_A_737866 and the family *Pasteurellaceae*.

The LDA value distribution histogram (Figure 4b) provides a quantitative comparison of the effect of each treatment on the gut microbiota, highlighting the most differentially abundant taxa with LDA scores above a certain threshold. The histogram displays the LDA scores for the taxa enriched in each group, reinforcing the findings from the cladogram. In the control group, taxa such as Firmicutes, *Bifidobacterium*_388775, *Bifidobacteriaceae*, *Turicibacteraceae*, and several others had higher LDA scores, indicating their significant presence in this group. The peppermint EO + thyme EO group showed significant enrichment of *Fusobacteriaceae* and *Fusobacteriales*, with higher LDA scores, suggesting the influence of EOs in promoting these taxa. The peppermint EO + feijoa peel extract group is characterised by higher LDA scores for the taxa *Rodentibacter*_A_734545 and *Pasteurellaceae*.

The lack of significant differences in overall microbial diversity among the treatment groups, from the taxonomic analysis, is corroborated by the cladogram and LDA histogram. While there are observable trends in the enrichment of specific microbial taxa in each treatment group, these differences are not statistically significant (*p* > 0.05). This implies that the administration of peppermint EO combinations, whether with thyme EO or feijoa peel extract, did not cause major changes in the gut microbiota composition.

While the present findings indicate no statistically significant changes in gut microbiota composition following administration of the synergistic combinations, these results should be interpreted with caution. The study was conducted over 28 days and employed a single rat model, which may not fully capture the potential longer-term or species-specific effects. In addition, although no significant shifts were detected using standard diversity and abundance metrics, the possibility of subtle functional or metabolic changes in the microbiome cannot be completely excluded. Future studies incorporating longer exposure times, animal models and metagenomic analysis would provide a more comprehensive understanding of the potential biological impacts. The absence of statistically significant differences does not necessarily imply the complete absence of biological effects. Rather, it indicates that any potential changes were not large enough to be detected within the experimental design and sample size employed. This distinction further supports the need for follow-up studies with expanded analytical sensitivity.

### 3.3. Histological Analysis

The histological findings provided insights into structural changes within the proximal colon following exposure to different synergistic combinations of EOs and extracts. The crypt depth and goblet cell per crypt are summarised in Table 3. Crypt depth and goblet cell counts serve as important indicators of mucosal integrity and secretory function, respectively [46].

**Figure 5:**
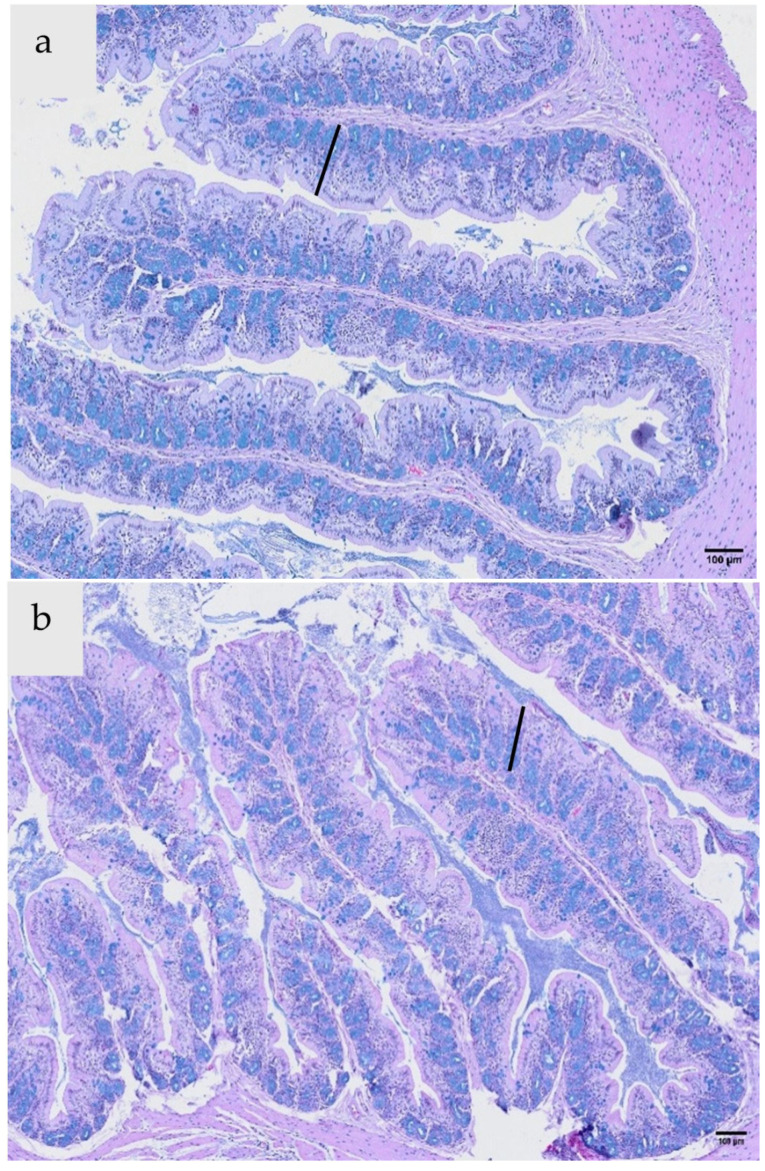

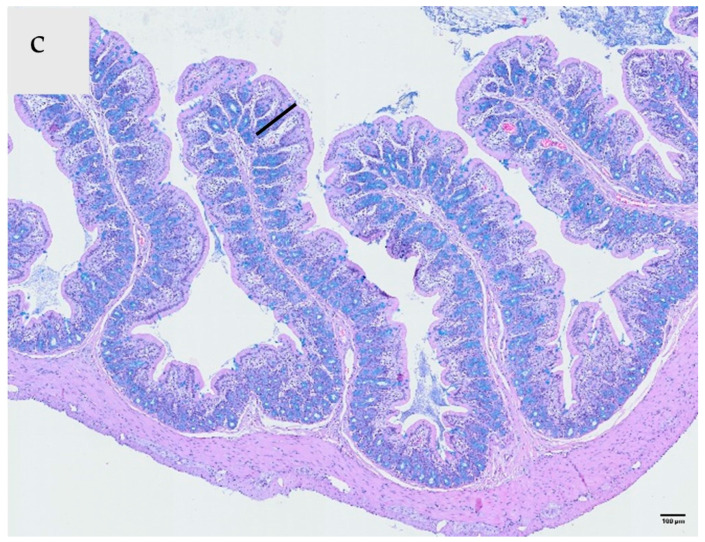
Microphotographs (20× magnification) of proximal colon sections from rats after 28 days of oral gavage with light olive oil (control) (**a**), peppermint + thyme (**b**), and peppermint + feijoa peel extract (**c**). The black line indicates crypt depth.

The colon crypt depth across all treatment groups was similar (Table 3 and Figure 5), demonstrating a consistent structural profile of the proximal colon, and no significant changes induced by the EOs and extract combinations. These findings are consistent with previous studies [1,25] that used plant-based additives in rats and pigs. However, the number of goblet cells per crypt in rats administered peppermint EO + feijoa peel extract was significantly lower (*p* < 0.05) compared to the other two treatment groups. While the reduced goblet cell density in the peppermint EO + feijoa extract group was statistically significant, there were no accompanying signs of mucosal damage or gut barrier disruption. Overall, the maintenance of crypt depth and goblet cell counts within normal ranges across the treatment groups is indicative of the safety profile of the EOs and extract combinations used in this study, and their minimal impact on the structural integrity of the proximal colon.

## 4. Conclusions

In conclusion, we found that the synergistic combinations of EOs and extracts, such as peppermint EO + thyme EO and peppermint EO + feijoa peel extract, did not elicit distinct shifts in microbial community structure compared to the control treatment. The taxonomic analysis and LEfSe analysis revealed that specific taxa were affected by these dietary interventions, and these potential microbial biomarkers should be targeted for further exploration. Elucidating the mechanisms underlying these dietary effects and their implications for host physiology will be crucial for advancing our understanding of gut microbiota-host interactions and developing personalised approaches for improving human health.

Further research on plant EOs influence on host-microbe interactions is required to better understand the overall impact on host health and well-being. The use of natural antimicrobials like EOs and extracts may offer an additional approach to suppress microbial proliferation and extend food product shelf life. Importantly, this approach aligns with current industry interest in reducing reliance on synthetic preservatives, particularly in minimally processed or ready-to-cook meat products where full cooking may not be uniformly achieved. While this study does not aim to replace cooking, it demonstrates the potential advantage of plant EO/extract-based treatments to enhance food safety.

## Figures and Tables

**Table 1:** Essential oil (EO) and extract doses were fed to rats in carrier oil via oral gavage.

	Control	Treatment Group 1 (Peppermint EO + Thyme EO)	Treatment Group 2 (Peppermint EO + Feijoa Peel Extract)
Peppermint EO		3.12 mg/g	3.12 mg/g
Thyme EO		3.12 mg/g	-
Feijoa peel extract		-	3.12 mg/g
Carrier oil (light olive oil)	1 mL	1 mL	1 mL

**Table 2:** Body weight of rats fed synergistic combinations of peppermint EO + thyme EO and peppermint EO + feijoa peel extract at a concentration of 3.12 mg/g.

	**Initial Body Weight**	**Final Body Weight**	**Weight Gain**
Control	242.9	373.0	130.0
Peppermint + Thyme	230.9	369.0	138.0
Peppermint + Feijoa peel extract	245.6	401.0	155.0
Standard error of the mean	12.4	29.6	18.0
*p* value	0.673	0.710	0.608

**Table 3:** Colon morphology of rats fed synergistic combinations of peppermint EO + thyme EO and peppermint EO + feijoa peel extract.

	Crypt Depth (µm)	Goblet Cells/Crypt
Control	260.4	17.85 ^b^
Peppermint + Thyme	286.6	17.61 ^b^
Peppermint + Feijoa peel extract	262.6	16.22 ^a^
Standard error of the mean	18.8	0.468
*p* value	0.555	0.003

Mean values with a different letter differ significantly, *p* < 0.05.

## Data Availability

The raw data supporting the conclusions of this article will be made available by the authors upon reasonable request.

## Abbreviations

The following abbreviations are used in this manuscript:

EOs	Essential oils
EO	Essential oil
PP	Peppermint
FjPlEx	Feijoa peel extract
PERMANOVA	Permutational multivariate analysis of variance
HSD	Honestly significant difference
PCoA	Principal coordinate analysis
LDA	Linear discriminant analysis
